# Incorporation of the magnetic field in GROMACS: validation and applications in biological systems†

**DOI:** 10.1039/d5ra00836k

**Published:** 2025-03-05

**Authors:** Diego Fernando Nieto-Giraldo, José Mauricio Rodas Rodríguez, Javier Ignacio Torres-Osorio

**Affiliations:** a Department of Chemistry, Universidad de Caldas Calle 65 # 26-10 Manizales Colombia diego.nieto@ucaldas.edu.co +57-6-8781501 +57-6-8781501; b Grupo de investigación en Magnetobiología, Department of Physics, Universidad de Caldas Calle 65 # 26-10 Manizales Colombia

## Abstract

The field of magnetobiology is garnering increasing interest due to its significant contributions across various disciplines, including biotechnology, medicine, and agriculture. Despite experimental evidence indicating the impact of magnetic fields on living organisms, the precise molecular-level effects of these fields remain unclear. Experimental studies of these phenomena at the molecular scale present significant challenges. In this regard, contributions from physics and theoretical chemistry are particularly relevant. However, the computational methodologies developed thus far are unable to incorporate magnetic fields into complex systems such as membrane proteins or biomolecules. In this context, the present work integrates the homogeneous magnetic flux density (*B*) term into the Verlet velocity algorithm implemented in the GROMACS package. This modification enables molecular dynamics simulations for such systems under the influence of a magnetic field. The implementation has been validated using two model systems: a free ion exposed to *B* ranging from 80 kT to 1500 kT, and a water box exposed to *B* between 0 T and 10 T. Furthermore, the stability of a protein was tested under the influence of *B* ranging from 0 T to 10 kT. The results demonstrated that the systems behaved in accordance with both theoretical and experimental expectations, thereby validating the modification of the algorithm and paving the way for future applications.

## Abstract 2

Validated MD simulations under magnetic fields provide insights into ion, water, and protein behavior, offering a valuable tool to advance understanding of magnetoreception mechanisms in biological systems.

## Graphical abstract

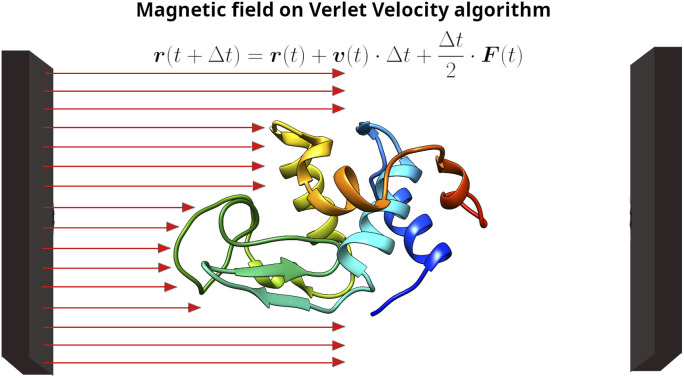

## 1 Introduction

The Earth's magnetic field is an intrinsic component of the environment for all living organisms. Any alteration in the magnetic flux density to which an organism is subjected has the potential to affect cellular processes.^1^ In this context, an alternative method for modifying magnetic flux density and obtaining specific benefits is through the application of exogenous magnetic fields generated by sources such as permanent magnets or electromagnets.

Currently, the magnetic treatment of biological systems has applications in various fields, including agriculture, medicine, and biotechnology.^2–8^ Magnetic fields have been shown to influence a multitude of cellular processes, including protein synthesis, enzymatic activity, nutrient uptake, and even the composition of biomolecules.^9–14^ Understanding the biochemical and biophysical mechanisms affected by exogenous magnetic fields is therefore essential. Despite their potential applications, there is still a lack of clarity regarding the impact of these fields on molecular-level processes from both theoretical and experimental perspectives.^14^

Grasping the modified mechanisms at the molecular level poses a significant experimental challenge; hence, computational methodologies can provide valuable theoretical insights and explanations. Molecular dynamics, a widely used methodology in the study of biomolecules, stands out as an effective approach as it allows for the analysis of interactions between particles and their movements. However, most molecular dynamics programs do not account for the influence of magnetic forces on particle motion and interaction. Even though some molecular dynamics programs have incorporated the effect of a magnetic field, such as in ^15^ where this functionality was implemented in GROMACS, the modified version is not publicly available.

In this context, our study proposes a modification to the Verlet velocity algorithm within the GROMACS package, incorporating a term for magnetic flux density (*B*). This modification enables molecular dynamics simulations for various systems under the influence of a magnetic field. The implementation was validated using two model systems: a free ion and a water box. Furthermore, the stability of a protein under the influence of the magnetic field was assessed to enhance our understanding of magnetoreception mechanisms in molecular systems and to verify the adequate behavior of the biomolecule concerning its conformational and energetic dynamics in the presence of a magnetic field.

The proposed modification to GROMACS facilitates the integration of magnetic forces into the time evolution of simulations, providing researchers with a valuable tool for exploring molecular interactions in biological systems. Moreover, this tool contributes to a deeper understanding of the effects of magnetic induction at the molecular level on physiological processes.

## 2 Methodology

### 2.1 Incorporation of *B* in the Verlet velocity algorithm in GROMACS

To incorporate *B* into the molecular dynamics simulations, the Verlet velocity algorithm implemented in the GROMACS package was modified. This algorithm updates the position (eqn (1)) and velocity (eqn (2)) at each molecular dynamics step in response to the forces experienced by all particles in the system.1
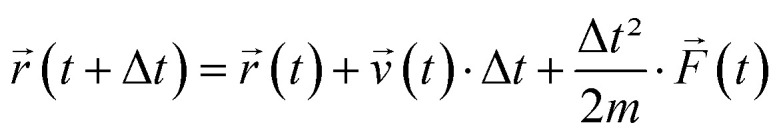
2
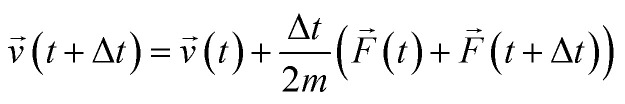


In this instance, the magnetic force was integrated according to the methodology proposed by Spreiter & Walter in 1999, as shown in eqn (3) and (4). In these equations, 
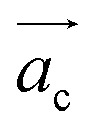
 represents the accelerations of the particles at the beginning of each integration step, while 
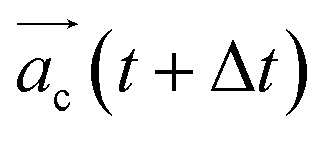
 corresponds to the accelerations at the end of that step.^16^3
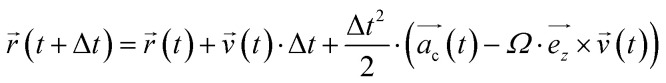
4
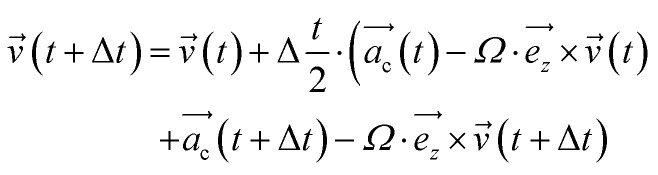


Here, the symbol *Ω* represents the Larmor frequency, expressed as *Ω* = *qB*/*m*. This incorporates the magnetic flux density (*B*) to which the system is subjected, along with the electric charge (*q*) and the mass of the particle (*m*) interacting with the magnetic field. Moreover, the unit vector denoted by 
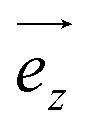
 indicates that the force is applied exclusively in the *z*-dimension. The incorporation of the magnetic field into GROMACS required implementing eqn (3) and (4) for each coordinate. Following the approach described in ref. 16, we implemented the inversion of 
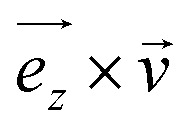
, as can be observed in the ESI.†

### 2.2 Modified code validation

To ensure the numerical validation of the modification, two systems with established behavior under the influence of a magnetic field were selected as references: a free ion and a water box.

#### 2.2.1 Electrically charged particle

The motion of a free ion (Na^+^ and Cl^−^) under the influence of *B* at 80 kT, 200 kT, 400 kT, 800 kT, and 1500 kT was simulated in a canonical NVE ensemble. Each simulation was executed for a total duration of 20 ps, with a time step of 0.1 fs. The resulting trajectories were characterized in terms of their radius and period of ion rotation.

#### 2.2.2 Water box system

The second model system comprises a box containing 887 water molecules of the SPC/E model, in the presence of a homogeneous *B* with values ranging from 0 T to 10 T. These simulations were conducted using an NVT ensemble at 300 K, employing the Nose–Hoover thermostat, and at a pressure of 1 bar over a 4 ns production period. The obtained trajectories were analyzed using the Diffusion Coefficient Tool implemented in VMD, which allows for the calculation of the self-diffusion coefficient of water.^18,19^

#### 2.2.3 Potential applications: lysozyme in water

Moreover, the modification was tested in a system comprising a biomolecule. However, the impact of magnetic fields on molecular processes in biological systems remains to be elucidated, particularly regarding the role of molecular dynamics. The current research presents a conformational and energetic analysis of the dynamics of lysozyme (PDB code: 1AKI^17^) in water under the influence of a magnetic field, using the GROMOS96 53a6 force field for the protein. This analysis aims to corroborate the hypothesis that there are no abrupt variations in the velocity or position of the atoms, which could lead to the breakdown of the simulation box or to anomalous behaviors in systems containing biomolecules.

**Fig. 1:**
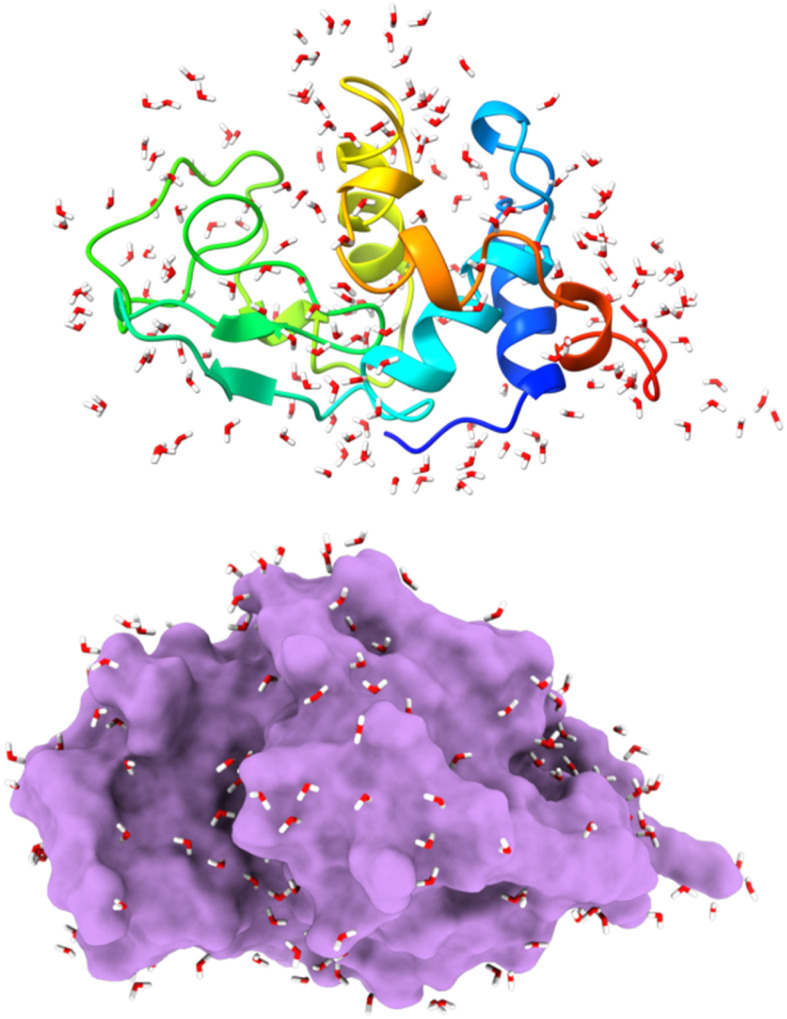
System with a lysozyme in water (PDB code: 1AKI^17^), with the protein surrounded by water molecules.

Fig. 1 depicts lysozyme contained in a water box, a system in which conformational variations of the protein were considered in response to *B* values ranging from 1 mT to 10 kT. For all systems, an equilibration process was conducted in an NVT ensemble for 0.5 ns, maintaining a constant temperature of 300 K using the Nose–Hoover thermostat. Subsequently, a further equilibration step of 0.5 ns was performed in an NPT ensemble, where the pressure was kept constant at 1 bar using the Parrinello–Rahman barostat.

During the aforementioned equilibration stages, a harmonic force constant of 1 kJ mol^−1^ nm^−1^ was applied to restrain the protein motion in all three dimensions. This was necessary to avoid any unwanted conformational changes that might have occurred due to density variations during the equilibration process. Upon completion of the equilibration stage, the constraints on protein motion were removed, and a 1 ns simulation was performed for each system under the influence of *B*, with values ranging from 1 mT to 1 MT. The *B* was incorporated from the NVT ensemble equilibration.

The results of these simulations were analyzed at the conformational level using root mean square deviation (RMSD). Notably, high variations in the RMSD value (above 2 Å) indicate that the protein undergoes conformational changes, which may be due to denaturation of its structure or conformational alterations that could affect its function.

Finally, to analyze these conformational variations, the radius of gyration *R*_g_ was considered. This is a commonly used measure in protein systems, as it characterizes the distribution of protein atoms around the center of mass. The value of *R*_g_ can estimate the degree of compactness of the protein, defined as the density of the atom distribution around the center of mass.

## 3 Results and analysis

### 3.1 Numerical validation of magnetic field incorporation in Verlet velocity

#### 3.1.1 Free ion system

**Fig. 2:**
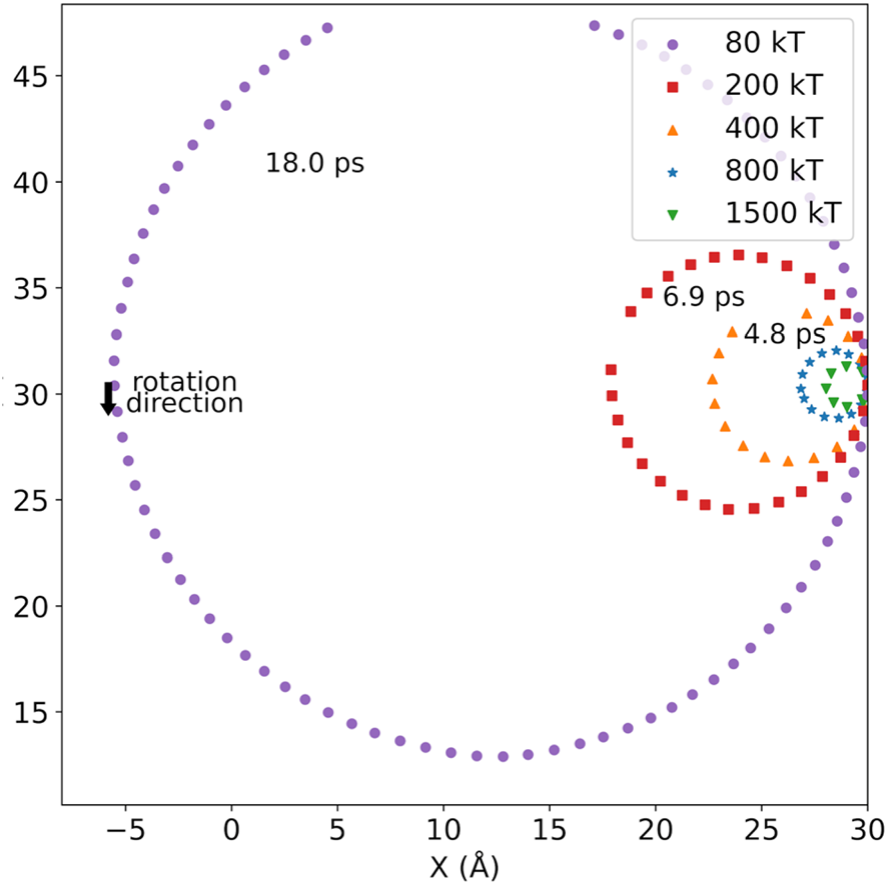
Circular trajectory of the sodium ion in the *xy* plane when subjected to *B* (80 kT, 200 kT, 400 kT, 800 kT and 1500 kT). The rotation period for some of the circular trajectories are also presented.

**Fig. 3:**
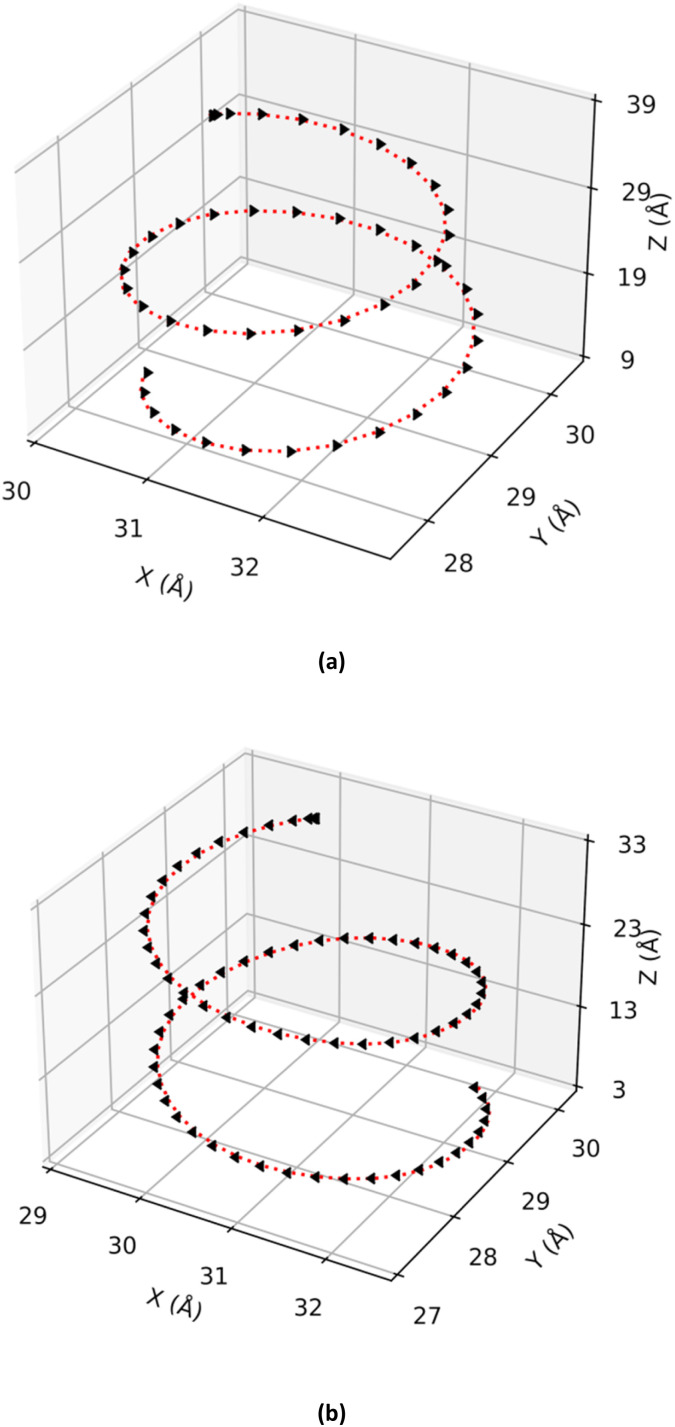
Helical trajectory of a chloride ion (a) and a sodium ion (b) under the influence of *B* of 1500 kT.

The motion of a free ion under the effect of a magnetic field is well documented in the literature.^20,21^ Therefore, the Na^+^ and Cl^−^ ions were selected as models to validate the correct implementation, and their motion can be visualized in Fig. 2 and 3. It can be observed that the sodium ion describes a circular motion in the *xy* plane under different values of *B* (Fig. 2), as expected for such a system. Additionally, both ions exhibit a helical trajectory, maintaining a constant velocity along 
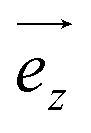
, which is characteristic of a charged particle moving in a magnetic field (Fig. 3).

It is also noteworthy that the direction of displacement of the particle depends on its electric charge. The chloride ion exhibits a clockwise rotation (Fig. 2a), while the sodium ion displays a counter-clockwise rotation (Fig. 2b). In both cases, *B* exerts its influence solely on the plane perpendicular to the vector indicating the direction of *B*, *i.e.*, the *z*-axis. This behavior aligns with theoretical expectations.

The trajectories were characterized numerically by the radius and period of rotation as a function of *B*, as summarized in Table 1. Both properties are inversely related to *B*. These findings corroborate existing theory, confirming the capability of this GROMACS implementation to accurately simulate the behavior of charged particles under varying magnetic fields.

**Table 1:** Radius and period of rotation of the sodium ion exposed to *B*

*B* (kT)	*r* a (Å)	*T* b (ps)
80	17.00	18
200	5.993	6.9
400	3.601	4.8
800	1.580	2.5
1500	0.9780	1.4

a *r*: path radius.

b *T*: rotation period.

#### 3.1.2 Water box system

The second model system evaluated numerically to validate the implementation consisted of a water box subjected to a magnetic field, the behavior of which has been previously studied by multiple authors in the presence of magnetic fields ranging from 0 T to 10 T.^21–25^

**Fig. 4:**
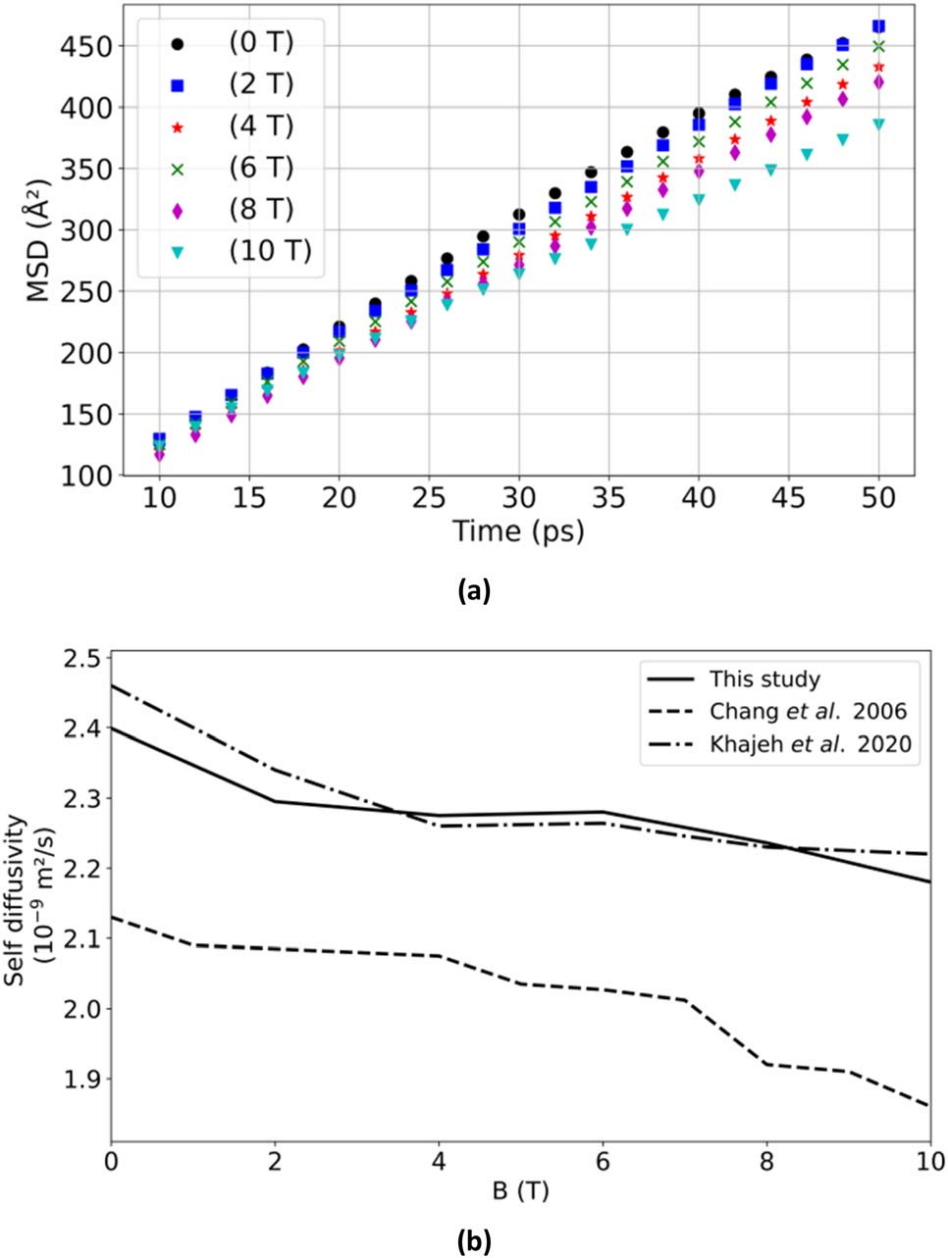
Characterization of the system composed of water molecules. (a) Mean square displacement of water as a function of time (MSD(*t*)) for the *B* values used. (b) Self-diffusion coefficient of water as a function of *B* in comparison to the value obtained by Chang *et al.*, in 2006 and by Khajeh *et al.*, in 2020.^21,25^

Fig. 4 depicts the root mean square displacement of water as a function of simulation time (MSD(*t*)), wherein the self-diffusion coefficient of water, *D*_w_, a measure of the rate at which water molecules diffuse in an aqueous medium, is derived from the linear rate of change of MSD with respect to time. Moreover, it is observed that an increase in *B* between 0 T and 10 T results in a gradual decrease in *D*_w_ (see Fig. 4b). This trend is consistent with findings reported in the scientific literature.^21,23,25^ This may be attributed to an increase in intermolecular interactions, which correlates with an enhanced structuring of the water molecule pool in response to magnetic induction.^25^ These observations illustrate that the implementation is capable of reproducing the behavior of a water box under magnetic flux density, a crucial consideration in molecular dynamics simulations of biological interest, as solvation in a water box is often essential.

### 3.2 Effect of *B* on a water solvated lysozyme

To investigate the impact of *B* on the potential energy and conformational dynamics of a water-solvated lysozyme, simulations were conducted across a range of field strengths from 0 T to 1 MT. Notably, at the highest field intensity (1 MT), the system underwent significant instability, leading to protein denaturation and expansion of the simulation box across all dimensions. This behavior reflects the excessive increase in particle velocity induced by such a high magnetic dosage, which disrupts the simulation properties.

**Fig. 5:**
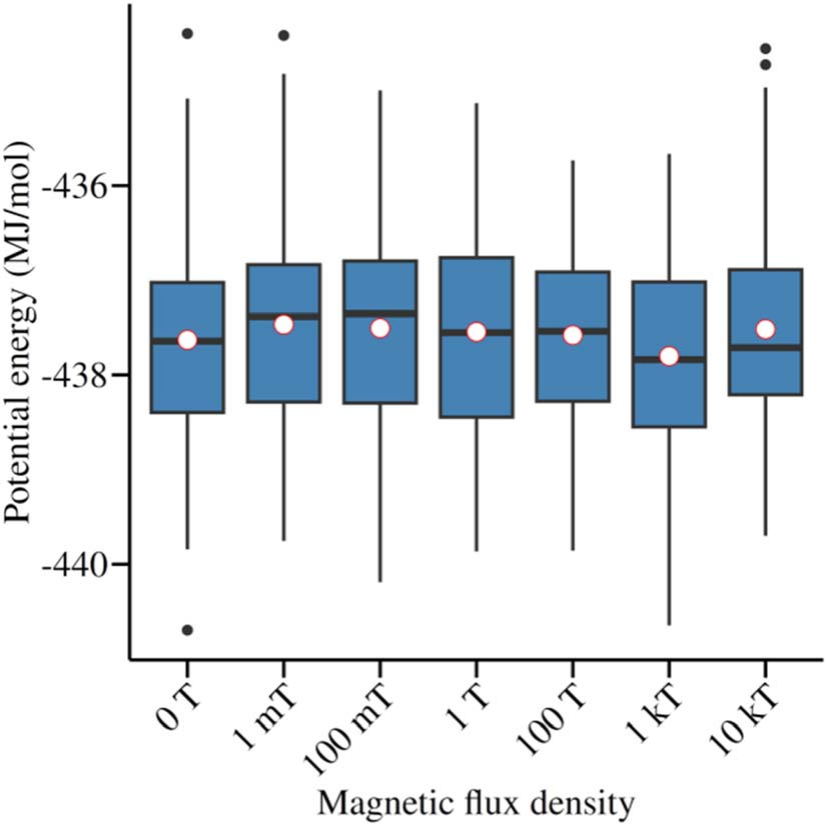
Variation of the potential energy of the system over the simulation time. Each data series corresponds to a magnetic flux density value as indicated in the legend.

**Fig. 6:**
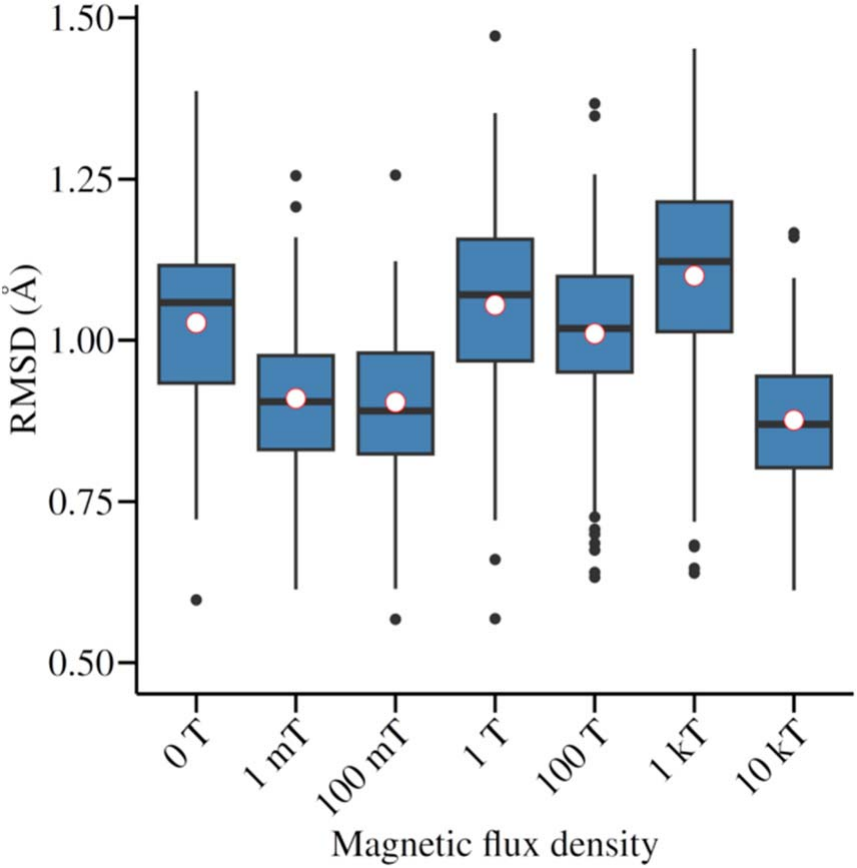
RMSD of the conformational dynamics of lysozyme in relation to the experimentally obtained structure.

With regard to the energetic dynamics of the system at varying *B*, it can be observed that there are no sudden changes in the energy of the systems exposed to *B* compared to the system that was not exposed, as illustrated in Fig. 5. This stability indicator is further corroborated by analyzing the conformational dynamics, which were evaluated through two parameters: the root mean square deviation (RMSD) and the radius of gyration (*R*_g_). For the former, the experimentally obtained conformation reported in 1AKI was used as a reference, resulting in RMSD values consistently below 1.5 Å. This confirms the low conformational variability (see Fig. 6).

**Fig. 7:**
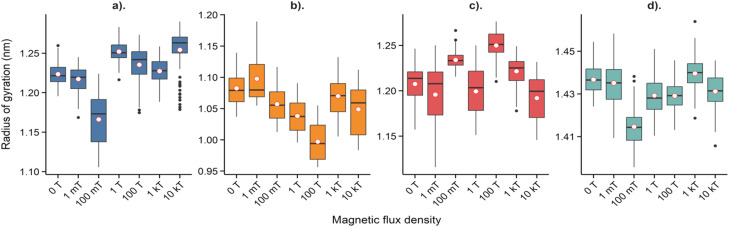
Radius of gyration for lysozyme under exposure to *B*. (a) Radius of gyration in *x*, (b) radius of gyration in *y*, (c) radius of gyration in *z*, (d) net radius of gyration.

The radius of gyration was also evaluated (see Fig. 7), with variations of less than 0.2 nm obtained. These results align with those of the previous evaluation and support the stability of the system under the influence of the evaluated *B* values. Nevertheless, in the *xy* plane, greater fluctuations are observed in the radius of gyration, indicating higher conformational variability in the plane affected by the magnetic field.

Although there is no experimental data to validate the results presented here, the behavior observed for a system such as lysozyme instills confidence in the implementation of the simulation of biomolecules under the effect of a magnetic field. This is evidenced by the absence of abrupt conformation changes throughout the simulation time, as well as the lack of artifacts, such as bubbles in the water box, and drastic changes in important parameters in molecular dynamics, such as temperature, pressure, and density of the system.

## 4 Conclusions

In the present work, the Verlet velocity algorithm implemented in the GROMACS package was modified to include a magnetic flux density term. The modified algorithm was then validated numerically with two model systems: the movement of a charged particle and a box of water. In both cases, there is experimental data on the behavior of these systems under the effect of magnetic fields. The results of the numerical validation demonstrated that the expected dynamics were reproduced, thereby validating the modification of the algorithm.

To ascertain the stability of a biomolecule under the influence of a magnetic field, a simulation was conducted on lysozyme in water. The simulation revealed that, throughout the duration of the experiment, the biomolecule's conformation exhibited no abrupt changes in terms of potential energy, root mean square deviation (RMSD), and radius of gyration. With the exception of 1 MT, which can be attributed to the high magnitude of *B*, these findings illustrate the potential of the present implementation in GROMACS for future molecular dynamics studies that consider the impact of a magnetic field. This also paves the way for a new tool in magnetobiology studies.

## Data availability

The data that support the findings of this study are available in the ESI.†

## Author contributions

The manuscript was written through the contributions of all authors.

## Conflicts of interest

The authors declare that there are no conflicts of interest.

## Supplementary Material

RA-015-D5RA00836K-s001
