# Supplementary material for: Incorporation of the magnetic field in GROMACS: validation and applications in biological systems
Source: RSC Adv. 2025 Mar 5;15(9):7121–6. doi: 10.1039/d5ra00836k (PMC11881925; doi:10.1039/d5ra00836k)
Supplement: RA-015-D5RA00836K-s001 [file RA-015-D5RA00836K-s001.pdf]

Supplementary Information (SI) for RSC Advances.
This journal is © The Royal Society of Chemistry 2025

Supplementary Information (SI) for RSC Advances.
This journal is © The Royal Society of Chemistry 2025.

Supporting information

Incorporation of the magnetic field in GROMACS: validation and applications in biological systems

Diego Fernando Nieto-Giraldo*[a], José Mauricio Rodas Rodríguez[a], and Javier Ignacio Torres-Osorio[b]

[a] *Department of Chemistry, Universidad de Caldas, Calle 65 # 26-10, Manizales, Colombia.*
[b] *Grupo de investigación en Magnetobiología, Department of Physics, Universidad de Caldas, Calle 65 # 26-10, Manizales, Colombia.* **Corresponding author: diego.nieto@ucaldas.edu.co*

1. Implemented equations of velocity for the incorporation of the magnetic field.

$$v_x(t+\Delta t) = \frac{1}{1+4(\Omega\Delta t)^2}\left[v_x(t) + \frac{1}{2}\Delta t\left(a_x(t) + a_x(t+\Delta t) + 2\Omega v_y(t)\right)\right] \\ + \frac{1}{4}(\Delta t)^2\Omega\left[a_y(t) + a_y(t+\Delta t) - \Delta v_x(t)\right]) \tag{1}$$

$$v_y(t+\Delta t) = (\text{like (1), exchange } x \leftrightarrow y,\ \text{replace } \Omega \to -\Omega) \tag{2}$$

$$v_z(t+\Delta t) = v_z(t) + \frac{1}{2}\Delta t\left[a_z(t) + a_z(t+\Delta t)\right]) \tag{3}$$

2. Implemented equations of position for the incorporation of the magnetic field.

$$r_x(t+\Delta t) = r_x(t) + \Delta t v_x(t) + \frac{1}{2}(\Delta t)^2\left[a_x(t) + \Omega v_y(t)\right]) \tag{4}$$

$$r_y(t+\Delta t) = (\text{like (4), exchange } x \leftrightarrow y,\ \text{replace } \Omega \to -\Omega \tag{5}$$

$$r_z(t+\Delta t) = r_z(t) + \Delta t v_z(t) + \frac{1}{2}(\Delta t)^2 a_z(t)) \tag{6}$$

3. Modified GROMACS code.

The modification of the GROMACS code were implemented on the file .../src/gromacs/modularsimulator/propagator.cpp, specifically, the functions updatePositions and updateVelocities were modified as follows:

A. Original code, line 117

```
...
v[a][d] += f[a][d] * invMassPerDim[a][d] * dt;
...
```

Modified code

```
...
float q = mdAtoms->mdatoms()->chargeA[a];
float larmor = 0;
if (d != 2)
{
        larmor = 0.0000965 * invMassPerDim[a][d] * q;
        float larmor_dt = larmor * dt;
        float v0 = v[a][0];
        float v1 = v[a][1];
        float f0 = f[a][0];
        float f1 = f[a][1];
        float inv_denom = 1.0 / (1 + larmor_dt * larmor_dt);
    if (d == 0)
    {
       v[a][0] = inv_denom * (v0 + dt * (f0 * invMassPerDim[a][0] + 2 * larmor
          * v1) + dt * dt * larmor * (f1 * invMassPerDim[a][0] - larmor * v0)
          );
    }
    else if (d == 1)
    {
       v[a][1] = inv_denom * (v1 + dt * (f1 * invMassPerDim[a][1] - 2 * larmor
          * v0) - dt * dt * larmor * (f0 * invMassPerDim[a][1] + larmor * v1)
          );
    }
}
else
{
    v[a][2] += f[a][2] * invMassPerDim[a][2] * dt;
}
...
```

B. Original code, line 134

```
...
xprime[a][d] = x[a][d] + v[a][d] * dt;
...
```

Modified code

```
...
float q = mdAtoms->mdatoms()->chargeA[a];
const ArrayRef<const RVec> invMassPerDim = mdAtoms->mdatoms()-> invMassPerDim;
float larmor0 = 0.0000965 * q * invMassPerDim[a][0];
float larmor1 = 0.0000965 * q * invMassPerDim[a][1];
float delta_x = x[a][0] + v[a][0] * dt;
xprime[a][0] = delta_x + dt * dt * 0.5 * (larmor0 * v[a][1]);
delta_x = x[a][1] + v[a][1] * dt;
xprime[a][1] = delta_x + dt * dt * 0.5 * (-larmor1 * v[a][0]);
delta_x = x[a][2] + v[a][2] * dt;
xprime[a][2] = delta_x;
...
```
